# Engagement in Care and Sexually Transmitted Infection Screening Among Patients Prescribed Doxycycline Postexposure Prophylaxis at a Midwestern HIV/Preexposure Prophylaxis Clinic

**DOI:** 10.1093/ofid/ofag251

**Published:** 2026-05-06

**Authors:** Vanessa Lo, Kathleen Nguyen, Sydney Bernhard, Joshua P Havens, Sara H Bares, Jennifer M Davis, Kimberly K Scarsi, Shawnalyn W Sunagawa

**Affiliations:** College of Pharmacy, University of Nebraska Medical Center, Omaha, Nebraska, USA; College of Pharmacy, University of Nebraska Medical Center, Omaha, Nebraska, USA; College of Pharmacy, University of Nebraska Medical Center, Omaha, Nebraska, USA; College of Pharmacy, University of Nebraska Medical Center, Omaha, Nebraska, USA; Division of Infectious Diseases, University of Nebraska Medical Center, Omaha, Nebraska, USA; Division of Infectious Diseases, University of Nebraska Medical Center, Omaha, Nebraska, USA; Division of Infectious Diseases, University of Nebraska Medical Center, Omaha, Nebraska, USA; College of Pharmacy, University of Nebraska Medical Center, Omaha, Nebraska, USA; Division of Infectious Diseases, University of Nebraska Medical Center, Omaha, Nebraska, USA; College of Pharmacy, University of Nebraska Medical Center, Omaha, Nebraska, USA

**Keywords:** doxycycline, postexposure prophylaxis, sexually transmitted infections

## Abstract

Our retrospective review included 186 patients who received a doxycycline postexposure prophylaxis (doxy-PEP) prescription, with 56% missing ≥1 quarterly sexually transmitted infection (STI) screening visit. Of attended visits, 63% included guideline-recommended multisite STI screenings. As doxy-PEP use grows, improving patient engagement is essential to ensure long-term effectiveness.

## Graphical Abstract

**Graphical Abstract:**
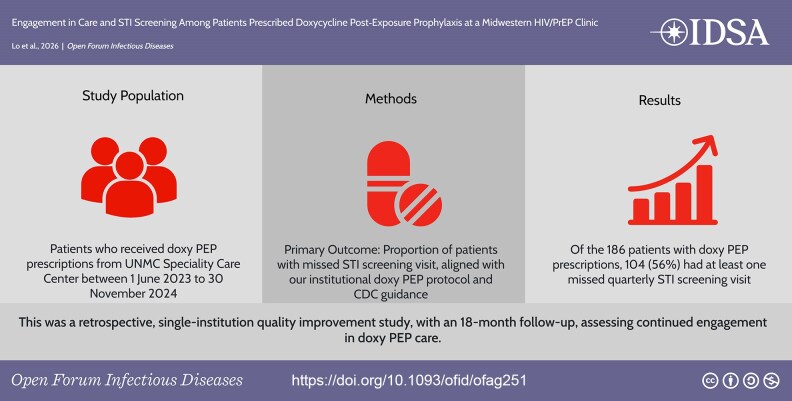
This graphical abstract is also available at Tidbit: https://tidbitapp.io/tidbits/engagement-in-care-and-sti-screening-among-patients-prescribed-doxycycline-post-exposure-prophylaxis-at-a-midwestern-hiv-prep-clinic-5b98c5b3-d907-4a45-86e3-482a0c6d0186?utm_campaign=tidbitlinkshare&utm_source=ITP

In June 2024, the Centers for Disease Control and Prevention (CDC) published clinical guidelines for the use of doxycycline postexposure prophylaxis (doxy-PEP), based on the results of key studies that demonstrated the efficacy in reducing the incidence of bacterial sexually transmitted infections (STIs) caused by *Neisseria gonorrhoeae* (GC), *Chlamydia trachomatis* (CT), and *Treponema pallidum* (syphilis) [1–5]. Through shared decision making, CDC guidelines recommend that doxy-PEP be prescribed as part of a comprehensive sexual health approach, including routine screening for CT, GC, and syphilis every 3–6 months, with testing at all anatomic sites of exposure [1]. Following these guidelines and earlier interim CDC guidance, the multidisciplinary team at the University of Nebraska Medical Center Specialty Care Center (UNMC SCC) clinic, including physicians, advanced practice providers, pharmacists, and nurses, implemented a doxy-PEP protocol for both patients receiving human immunodeficiency virus (HIV) care and those receiving preexposure prophylaxis (PrEP) services [6]. After implementation, we assessed our initial 9-month clinic utilization and found nonadherence to clinic protocol with 30% of doxy-PEP prescriptions, largely due to missed STI screenings at the initial visit [6]. With missed STI screenings in patients using doxy-PEP, concerns have also been raised about potential antimicrobial resistance [7, 8]. Thus, we assessed patient engagement at UNMC SCC by analyzing the number of missed STI screening visits among patients prescribed doxy-PEP.

## METHODS

As a quality improvement project, per SQUIRE (Standards for QUality Improvement Reporting Excellence) guidelines [9], we retrospectively evaluated patients who received doxy-PEP prescriptions from UNMC SCC between 1 June 2023 and 30 November 2024 and had ≥12 months of follow-up after the initial doxy-PEP prescription. Patients receiving doxycycline for other indications, such as treatment of STIs or dermatologic conditions, were excluded. The primary outcome was the proportion of patients with missed STI screenings, defined as <4 STI screening visits in 12 months. This threshold is based on our clinic protocol, which operationalized CDC doxy-PEP guidance of screening every 3–6 months as quarterly screening for all patients, including those receiving HIV care, given the novelty of doxy-PEP [1, 6]. Secondary outcomes included the proportion of patients with <2 STI screening visits in 12 months, the number of missed STI screening visits, guideline-recommended STI and HIV screenings conducted at every visit attended, STI diagnoses while doxy-PEP was prescribed, and doxy-PEP prescribed in concordance with clinic protocol at every visit attended. We defined concordance with clinic protocol as appropriate STI and HIV screenings. We performed descriptive statistical analysis for cohort characteristics and outcomes, using χ^2^ tests to assess associations and compare outcomes between people with HIV and those receiving HIV PrEP. All analyses were completed using SPSS software, version 29 (IBM), and differences were considered statistically significant at *P* < .05. Our institutional review board deemed this project exempt from review and as not including factors necessitating patient consent.

## RESULTS

In total, 186 patients received a doxy-PEP prescription during our study period with ≥12 months of follow-up data available. The majority were male (96.8%), and the mean age was 39 years. Approximately half of the patients were seen for HIV PrEP, and the other half were seen for HIV-associated care. Patients had a median of 1 STI (range, 0–7) in the 12 months before their initial doxy-PEP prescription. Additional cohort characteristics are listed in Table 1.

**Table 1.** Cohort Characteristics

Characteristic	Patients, No. (%)^a^ (N = 186)
Age, mean (SD), y	39 (10.8)
Sex/gender	
Male	180 (96.8)
Female^b^	1 (0.5)
Transgender male	5 (2.7)
Race	
White	125 (67.2)
Black	28 (15.1)
Asian	6 (3.2)
American Indian/Pacific Islander	1 (0.5)
Other	26 (14)
Non-Hispanic ethnicity	143 (76.9)
Reason for care at UNMC SCC	
HIV	102 (54.8)
PrEP	84 (45.2)
Insurance	
Uninsured/self-pay	118 (63.4)
Commercial/private	46 (24.7)
Government funded/ADAP	22 (11.8)
STIs in the 12 mo before initial prescription, median no. (range)	1 (0–7)
Provider ordering doxy-PEP	
Pharmacist	81 (43.5)
Advanced practice provider	54 (29)
Attending physician (MD/DO)	28 (15.1)
ID fellow	23 (12.4)

Abbreviations: ADAP, AIDS Drug Assistance Program (ADAP); doxy-PEP, doxycycline postexposure prophylaxis; HIV, human immunodeficiency virus; ID, infectious diseases; PrEP, preexposure prophylaxis; STI, sexually transmitted infection; UNMC SCC, University of Nebraska Medical Center Specialty Care Center.

^a^Data represent no. (%) of patients unless otherwise specified.

^b^UNMC SCC doxy-PEP protocol modeled per CDC guidelines, which does not recommend doxy-PEP in cisgender females; however, the female patient was initially prescribed doxy-PEP at an outside facility, but receivved follow-up care at UNMC SCC.

Of the 186 patients with a doxy-PEP prescription, 104 (56%) had ≥1 missed quarterly STI screening visit, with a median of 1 missed STI screening visit (range, 0–5). Of these 104 patients, 18 (10%) had attended <2 STI screening visits in a 12-month period. For the attended visits, 118 patients (63%) received STI screening and 175 (94%) received HIV testing/viral load monitoring at every visit; doxy-PEP was prescribed in concordance with clinic protocol at every visit in 110 patients (59%). In total, 27 patients (14.5%) had an STI diagnosed while being prescribed doxy-PEP, with the most common being GC (n = 16), followed by multiple STIs (n = 5), CT (n = 3), and syphilis (n = 3). In the univariate analyses, there was no significant difference in having ≥1 missed STI screening visit between patients receiving HIV-associated care and those receiving PrEP (*P* = .38), but patients receiving PrEP were less likely than those receiving HIV-associated care to receive appropriate STI screenings at every visit (*P* = .03). However, when it came to having an STI diagnosed while being prescribed doxy-PEP, there was no significant difference between patients with (n = 17) and those without (n = 10) a missed STI screening visit (*P* = .42). Additional univariate analyses are reported in Table 2.

**Table 2.** Univariate Analysis

Adherence to Screening and doxy-PEP	Patients, No. (%)		*P* Value
	Receiving HIV Care (n = 102)	Receiving PrEP (n = 84)	
≥1 Missed quarterly STI screening visit^a^	60 (59)	44 (52)	.38
Appropriate STI screening at every visit attended^b^	72 (71)	46 (55)	.03^c^
Appropriate HIV screening at every visit attended^d^	100 (98)	82 (98)	.84
doxy-PEP prescribed in concordance with protocol at every visit attended^e^	71 (70)	44 (52)	.02^c^

Abbreviations: doxy-PEP, doxycycline postexposure prophylaxis; HIV, human immunodeficiency virus; PrEP, preexposure prophylaxis; STI, sexually transmitted infection.

^a^Among patients with STIs diagnosed while doxy-PEP was prescribed, 17 were among the 104 patients with missed visits, and 10 were among the 82 with no missed visits (*P* = .42).

^b^Appropriate STI screening defined as screenings at all anatomic sites of exposure for *Neisseria gonorrhoeae*/*Chlamydia trachomatis*, per subjective patient report, and screening for syphilis.

^c^Significant at *P* < .05.

^d^HIV screening every 3 months for patients on PrEP and HIV viral load monitoring every 6–12 months for patients receiving HIV-associated care.

^e^Concordance with University of Nebraska Medical Center Specialty Care Center doxy-PEP protocol, defined as having both appropriate STI and HIV screenings at every visit.

## DISCUSSION

The continued effectiveness of doxy-PEP has been demonstrated in real-world settings, but there remains little insight into the sustained retention in care for patients prescribed doxy-PEP, which is necessary due to the recommended routine STI screenings [10–12]. In our cohort, more than half of patients prescribed doxy-PEP had ≥1 missed quarterly STI screening visit, defined as attending <4 STI screening visits in a 12-month period, with no difference between those receiving HIV-associated care or PrEP. However, the median number of missed STI screening visits occurring over 12 months was 1 (range, 0–5) and only 10% of patient did not attend ≥2 STI screening visits within a 12-month period, which is aligned with the CDC guideline recommendation to provide STI screenings every 3–6 months [1]. Given the median of only 1 missed STI screening visit annually, and given previous literature assessing less frequent STI screening in patients receiving PrEP, there is potential to individualize screening intervals for patients receiving doxy-PEP [13, 14]. There was also no difference in new diagnoses of STI while doxy-PEP was being prescribed between patients who had a missed STI screening visit and those who did not (Table 2); however, we acknowledge that in patients who missed STI screening visits there is the possibility that we missed a new STI diagnosis.

These engagement in care data combined with the previously reported effectiveness of doxy-PEP in preventing new incidence of STIs in real-world settings [6, 11, 12], suggest that our clinic's standardized protocol of screening patients for STIs every 3 months may be more intensive than required for some patients, and some patients may be appropriate for STI screenings every 6 months and/or when there is an acute concern. While this may complicate scheduling, aligning STI screening visits with other clinical visits could streamline patient care and further improve retention.

In addition, we found that there is still an opportunity to improve our clinic's completion of appropriate STI screenings, including GC/CT at all patient-reported anatomic sites of exposure and syphilis. In our cohort, only 63% of patients had appropriate STI screenings at every visit, with significantly fewer appropriate STI screenings in patients receiving PrEP (55%) than in those receiving HIV-associated care (71%). Although appropriate STI screening was emphasized and reinforced during protocol education, we hypothesize that screenings were missed in part because of patient concerns about screening-related costs, particularly given the high number of uninsured/self-pay patients in our cohort, or that patients deferred screenings because of little sexual activity in the preceding months. While our clinic currently has a partnership with our county health department to provide free GC/CT screenings at all anatomic sites of exposure, we are unable to offer free syphilis screenings. While our clinic's doxy-PEP utilization was not assessed in the current study, a previous review demonstrated that most missed STI screenings were for syphilis ^[6],^ further emphasizing the importance of patient access to affordable STI screening options.

This was a retrospective, single-institution quality improvement study with an 18-month follow-up to assess retention in doxy-PEP care. While these limitations may have decreased our ability to capture the full impact of doxy-PEP on continued engagement in care, this follow-up duration is aligned with previous data assessing patients lost to follow-up beyond 12 months after initiating PrEP [15]. While we could not extract causes for missed STI screening visits during this retrospective record review, reasons may include periods of sexual inactivity and/or a lack of new partners in preceding months. This limitation highlights an opportunity for clinicians to engage patients in proactive discussions regarding their specific doxy-PEP usage to personalize their STI screening frequency. Finally, our cohort population was majority male and white, which may limit overall generalizability, though it is reflective of our clinic's overall demographics.

Our study provides insight into engagement in care for patients prescribed doxy-PEP as well as challenges with continued engagement in care/attendance at routine STI screening visits in an HIV/PrEP clinic setting. Sustained engagement in care for patients receiving doxy-PEP is essential to ensure appropriate and timely STI screening, diagnosis, and insight into potential associated antimicrobial resistance. Our findings demonstrate the importance of monitoring/assessing patients’ engagement in care to ensure adherence to clinic and/or CDC guidelines for regular follow-up and monitoring and to support doxy-PEP utilization and sustained effectiveness in STI prevention.
